# JCAD/KIAA1462: biology, pathophysiology, and translational perspectives in vascular disease

**DOI:** 10.1093/cvr/cvag182

**Published:** 2026-08-25

**Authors:** Margherita Moriero, Simon Kraler, Amedeo Tirandi, Davide Di Vece, Thomas F Luscher, Federico Carbone, Davide Ramoni, Aldo Bonaventura, Giovanni G Camici, Fabrizio Montecucco, Stefano Ministrini, Yifan Wang, Luca Liberale

**Affiliations:** First Clinic of Internal Medicine, Department of Internal Medicine, University of Genoa, 6 viale Benedetto XV, Genoa 16132, Italy; Center for Molecular Cardiology, University of Zürich, Schlieren, Switzerland; Department of Cardiology, University Hospital of Bern, Bern, Switzerland; First Clinic of Internal Medicine, Department of Internal Medicine, University of Genoa, 6 viale Benedetto XV, Genoa 16132, Italy; Center for Molecular Cardiology, University of Zürich, Schlieren, Switzerland; First Clinic of Internal Medicine, Department of Internal Medicine, University of Genoa, 6 viale Benedetto XV, Genoa 16132, Italy; Department of Internal Medicine B, University Medicine Greifswald, Greifswald, Germany; Center for Molecular Cardiology, University of Zürich, Schlieren, Switzerland; Royal Brompton and Harefield Hospitals and Imperial College, London, UK; School of Cardiovascular Medicine and Sciences, Kings College London, London, UK; First Clinic of Internal Medicine, Department of Internal Medicine, University of Genoa, 6 viale Benedetto XV, Genoa 16132, Italy; AOM IRCCS Ospedale Policlinico San Martino, Genoa, Italy; First Clinic of Internal Medicine, Department of Internal Medicine, University of Genoa, 6 viale Benedetto XV, Genoa 16132, Italy; AOM IRCCS Ospedale Policlinico San Martino, Genoa, Italy; Medical Center, S.C. Medicina Generale 1, Ospedale di Circolo and Fondazione Macchi, Department of Internal Medicine, ASST Sette Laghi, Varese, Italy; Center for Molecular Cardiology, University of Zürich, Schlieren, Switzerland; Department of Research and Education, University Hospital Zurich, Zurich, Switzerland; First Clinic of Internal Medicine, Department of Internal Medicine, University of Genoa, 6 viale Benedetto XV, Genoa 16132, Italy; AOM IRCCS Ospedale Policlinico San Martino, Genoa, Italy; Center for Molecular Cardiology, University of Zürich, Schlieren, Switzerland; Center for Molecular Cardiology, University of Zürich, Schlieren, Switzerland; First Clinic of Internal Medicine, Department of Internal Medicine, University of Genoa, 6 viale Benedetto XV, Genoa 16132, Italy; AOM IRCCS Ospedale Policlinico San Martino, Genoa, Italy

**Keywords:** JCAD, KIAA1462, Vascular, Atherosclerosis, Ischemic stroke, Inflammation, Arterial thrombosis, Cardiovascular risk

## Abstract

Junctional cadherin 5-associated protein (JCAD/KIAA1462) is an endothelial junctional protein encoded by a locus robustly associated with coronary artery disease and myocardial infarction in genome-wide association studies. Initially identified as a structural component of endothelial cell–cell junctions, JCAD has since emerged as a mechanosensor and regulator of key intracellular pathways (i.e. Hippo/YAP, PI3K/Akt, and mitogen-activated protein kinase/extracellular signal-regulated kinase) with functional consequences for endothelial homeostasis, inflammation, and vascular tone. In this review, we summarize the current understanding of JCAD biology, from its molecular features and pathway interactions to its experimental roles in atherosclerosis, arterial thrombosis, and ischaemic stroke. We further discuss emerging translational evidence suggesting circulating JCAD as an independent predictor of adverse cardiovascular events in patients with acute coronary syndromes, and its prognostic potential in ischaemic stroke. The review also addresses the expanding biology of JCAD within the cardiovascular system, hypothesizing links to conditions, such as hypertension, heart failure with preserved ejection fraction, and calcific aortic valve disease, as well as key limitations and therapeutic challenges. Overall, the available evidence highlights JCAD as a biologically plausible and potentially clinically relevant endothelial mediator, though independent validation and substantial mechanistic work remain necessary before its translational potential can be fully realized.

## Graphical Abstract

**Graphical Abstract:**
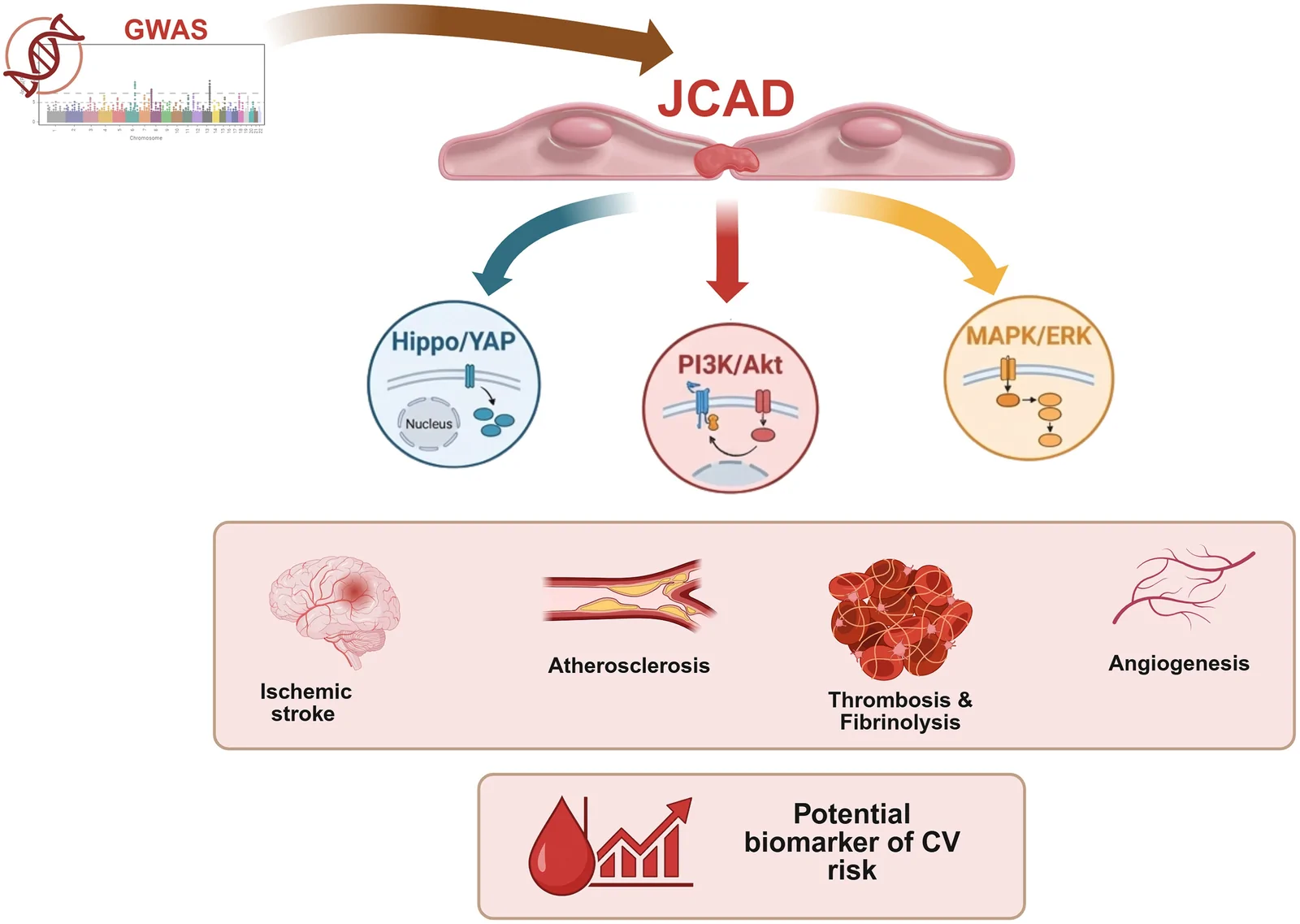
JCAD/KIAA1462 as an endothelial mediator of vascular disease. Genetic variants at the JCAD locus, identified through genome-wide association studies, associate with increased JCAD expression in arterial tissue and with risk of coronary artery disease and ischaemic events. JCAD modulates endothelial cell behaviour through three downstream signalling axes (i.e. Hippo/YAP, PI3K/Akt, and MAPK/ERK) contributing to atherosclerosis, arterial thrombosis, and acute ischaemic stroke, with distinct mechanistic contributions in each disease context. Elevated circulating JCAD levels associate with adverse cardiovascular outcomes and impaired fibrinolysis in patients with acute coronary syndromes, supporting its candidacy as a biomarker of residual thrombotic risk. Created in BioRender. Montecucco, F. (2026) https://BioRender.com/qt34rgs

**Time of primary review: 41 days**

## 1. Introduction

The identification of lipid- and inflammation-associated targets, such as PCSK9 and NLRP3 inflammasome-related cytokines, has spurred innovative clinical trials,^1^ however—despite their pharmacological modulation—the residual risk of both cerebro- and cardiovascular conditions remains substantially high, highlighting the need for novel molecular targets that can mechanistically explain residual risk and provide actionable therapeutic opportunities. Junctional cadherin 5-associated, also known as junctional protein associated with coronary artery disease (JCAD) or KIAA1462, is a 145 kDa phosphoprotein that is predominantly expressed by endothelial cells (ECs) throughout the cerebro- and cardiovascular system.^2^ Genetic variants of *JCAD* are linked to cardiovascular disorders, such as systemic arterial hypertension, atherosclerosis, and acute coronary syndromes.^3–6^ Experimentally, JCAD is implicated in tumour progression and metastatic invasion by promoting intra-tumour angiogenesis.^7–9^ A possible involvement of JCAD in neurodegenerative and inflammatory diseases has also been hypothesized.^10,11^ Here, we review the current knowledge about the biology of this novel protein and the most recent evidence for its pathophysiological role in cerebro- and cardiovascular conditions.

## 2. Molecular features and biological functions

### 2.1 Overview of JCAD biology

The KIAA1462 gene is located on chromosome 10p11.23, a genomic region encompassing several loci associated with morbid obesity.^12–14^ The nucleotide sequence of KIAA1462 encodes a protein consisting of 1359 amino acids, which is highly conserved among vertebrates. Although clearly defined functional domains remain to be characterized, a small segment, comprising 113 amino acids, exhibits homology to Rho-associated protein kinases (ROCK) 1 and 2, as well as with cingulin.^2,15^

ROCK 1 and 2 are serine/threonine kinases that function as downstream effectors of the guanosine-triphosphate (GTP)-binding proteins RhoA, RhoB, and RhoC. While Rho-kinases are ubiquitously expressed, the two isoforms (ROCK 1 and 2) exhibit distinct biological effects, cellular localization, and tissue-specific expression patterns. Both isoforms share a high degree of homology and are involved in various cellular processes, including cytoskeletal organization, cell motility, cell-to-cell adhesion, proliferation, and apoptosis.^16,17^ Notably, ROCK1 and ROCK2 display the highest homology in their N-terminal regions, where the kinase domain is situated.^18^

In the cardiovascular system, ROCKs play a critical role by negatively regulating nitric oxide (NO) production in ECs while promoting contraction in vascular smooth muscle cells. Given the multiple protective functions of NO, ROCK expression is associated with endothelial dysfunction, vascular hypertonia, and atherosclerosis.^19^ Furthermore, Rho-kinases are pivotal in various stages of tumorigenesis.^20^ The cytoskeletal rearrangements mediated by ROCKs confer resistance to apoptosis in oncogene-bearing myelopoietic cells, thereby facilitating their progression to leukaemia.^21^ Additionally, similar mechanisms may promote metastatic dissemination and immune modulation.^22,23^

Cingulin is an adaptor protein predominantly expressed in epithelial cells, where it mediates the interaction between the cytoskeleton and tight junctions.^24^ Similar to ROCKs, cingulin interacts with RhoA, functioning as an upstream modulator.^25,26^ This interaction implies that cingulin expression inhibits the activation of the RhoA/ROCK signalling pathway. Furthermore, cingulin promotes the transcription of the junctional component claudin-2^27^; intriguingly, RhoA acts as an inhibitor of claudin-2 transcription. This suggests that cingulin and the RhoA/ROCK signalling pathways represent two competitive signalling systems originating from the cytoskeleton.

Like ROCKs, the functional site of JCAD is likely located in the N-terminal region. This portion of JCAD contains a proline-rich region that binds to cadherin-5 (also known as vascular endothelial cadherin or VE-cadherin), facilitating its interaction with the cytoskeleton and forming a structure referred to as a ‘ruffling site’. This site is critical for initiating cellular movement through the interaction between the cytoskeleton and the membrane.^8^ Proteomic studies have suggested interactions between JCAD and actin-binding proteins, including Filamin C, cytoskeleton-associated protein 4 (CKAP4), and TRIO and F-actin binding protein (TRIOBP).^28^ Based on these findings, JCAD was postulated to function as a mechanosensor in ECs.^29^ The disruption of interactions between tight junctions and the cytoskeleton could represent a trigger for JCAD activation.^30^

### 2.2 Molecular pathways regulated by JCAD

Though our understanding on the intracellular pathways regulated by JCAD is largely incomplete, available evidence suggests that JCAD may interact with three key intracellular pathways *(Figure 1)*:

**Figure 1:**
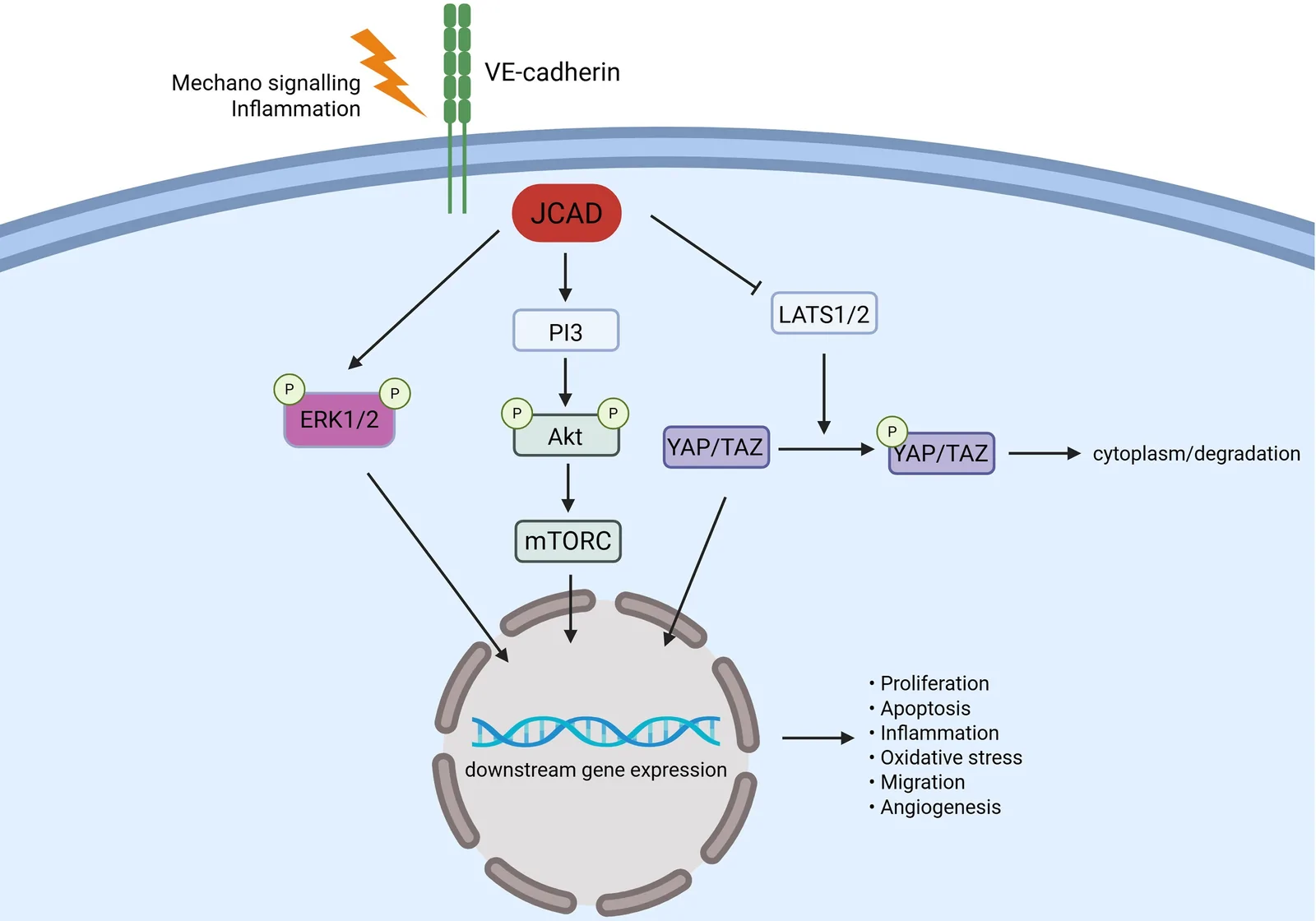
Molecular pathways regulated by JCAD. (1) Hippo pathway: JCAD inhibits Lats2-mediated phosphorylation of YAP, promoting its nuclear localization and transcription of anti-apoptotic and pro-inflammatory genes, contributing to atherosclerosis and coronary artery disease. (2) PI3K/Akt pathway: JCAD interacts with Akt, potentially inhibiting its phosphorylation and downstream activation of survival-promoting targets, such as mTORC1, FOXO, and IκB kinase. This effect has been linked to arterial thrombosis and blood-brain barrier damage following ischaemia/reperfusion. (3) MAPK pathway: JCAD knockdown reduces ERK 1/2 phosphorylation and VEGF receptor 2 signalling, though the exact molecular interaction remains unclear. ERK 1/2 activation promotes cell proliferation and survival by regulating transcription factors such as c-Fos, c-Myc, and c-Jun. Overall, JCAD emerges as a key modulator of endothelial cell function, inflammation, and vascular disease progression. JCAD, junctional Cadherin 5 associated; Lats2, large tumour suppressor kinase 2; YAP, Yes-associated protein; PI3K, phosphoinositide 3-kinase; Akt, protein kinase B; mTORC1, mechanistic target of rapamycin complex 1; FOXO, Forkhead Box O; IκB, inhibitor of nuclear factor kappa-B; MAPK, mitogen-activated protein kinase; ERK 1/2, extracellular signal-regulated kinases 1 and 2; VEGF, vascular endothelial growth factor. Created in BioRender. Montecucco, F. (2026) https://BioRender.com/qyiaxqt.

#### 2.2.1 Hippo pathway

This highly conserved signalling pathway is a main regulator of organ size, as it inhibits cell proliferation.^31^ Its name derives from one of the core components of the pathway, originally discovered in *Drosophila* flies. In humans, the core components of the pathway are the onco-suppressant kinases Mst1 and 2 (corresponding to Hippo in *Drosophila*), which associate with the WW-scaffolding domain protein WW45 and phosphorylate the Large tumour suppressors (Lats) 1 and 2.^32^ Lats1/2 phosphorylates the transcription co-activators Yes associated protein (YAP) and WW-domain-containing transcription regulator 1 (WWTR1 or TAZ) promoting their extrusion from the nucleus and terminating their transcriptional activity. Nuclear YAP/TAZ promote cell proliferation and inhibit apoptosis, so their cytosolic localization results in an inhibition of tissue growth.^33^ JCAD directly interacts with Lats2 and inhibits YAP phosphorylation, promoting its intranuclear localization and transcription of genes coding for anti-apoptotic and pro-inflammatory proteins.^9,15,34^ Such mechanism seems to mediate JCAD’s role in atherosclerosis and coronary artery disease.^15,29^

#### 2.2.2 Phosphoinositide 3-kinase (PI3k)/Akt

This ubiquitously expressed intracellular pathway regulates cell proliferation and survival by inhibiting apoptosis and promoting cell cycle progression.^35^ It also regulates inflammation, angiogenesis, autophagy as well as glucose, and lipid metabolisms. The role of JCAD as a negative regulator of the PI3K/Akt pathway has been demonstrated in arterial thrombosis and blood–brain barrier damage following ischaemia/reperfusion, where treatment with the PI3K/Akt inhibitor Wortmannin reversed the effect of JCAD silencing suggesting its causative role.^30,34^ With reference to atherosclerosis, JCAD silencing in ECs associated with reduced Akt phosphorylation in response to acute shear stress.^29^ PI3K is involved in the signal transduction of several transmembrane receptors.^36^ By phosphorylating the 3′ position of phosphatidylinositol, it activates a phosphorylation cascade that ultimately results in the phosphorylation of Akt on Thr308. To be fully activated, Akt needs a second phosphorylation in Ser473, which can be provided by the phosphatidylinositol-3,4,5, -triphosphate (PIP3) cascade, or by the mammalian target of Rapamycin complex 2 (mTORC2).^37^ In turn, phosphorylated Akt activates multiple substrates, including mTORC1, forkhead box protein O (FOXO), and IκB kinase, eventually inhibiting apoptosis.^38^ The pathway is regulated by intracellular phosphatases, such as the phosphatase and tensin homologue (PTEN), which terminates the signalling cascade by dephosphorylating PIP3. Mutations of these phosphatases result in a constitutive activation of the pathway.^39^ While phosphatase activity has not yet been attributed to JCAD, its direct interaction with Akt has been demonstrated. This interaction suggests that JCAD may inhibit Akt phosphorylation, potentially by sterically hindering access to the phosphorylation site through competitive or non-competitive binding mechanisms.^34^ In ECs, PI3K/Akt is a downstream pathway of vascular endothelial growth factor (VEGF), promoting survival and replication in these cells,^40^ and epithelial growth factor, which promotes resistance to hypoxia by inducing the hypoxia inducible factor 1α (HIF1α).^41^

#### 2.2.3 Mitogen-activated protein kinases

This term defines a group of three protein kinases, namely extracellular signal-regulated kinase (ERK) 1/2, c-Jun N-terminal kinase (JNK) and p38, which constitute the core of an intricated intracellular signalling pathway.^42^ Like Akt, ERK 1/2 is a serine-threonine protein kinase, downstream of the *Rattus* sarcoma (Ras) pathway.^43^ Ras is the second messenger of multiple growth factors constitutively bound to guanosine-diphosphate (GDP) in its inactive form. Once activated, Ras exchanges GDP with GTP, binds the rapid accelerated sarcoma (Raf) protein and starts a phosphorylation cascade.^44^  *Jcad* knock-down was associated to reduced ERK 1/2 phosphorylation and reduced signalling of the VEGF receptor 2 in ECs.^8^ The molecular interaction between JCAD and this signalling cascade is still unclear. However, phosphorylated ERK 1/2 activates multiple transcription factors, including c-Fos, c-Myc, and c-Jun, which inhibit apoptosis and promote cell proliferation and differentiation.^45^ It should be noted that, unlike the Hippo/YAP and PI3K/Akt axes, the mechanistic link between JCAD and the mitogen-activated protein kinase/ERK pathway remains preliminary and requires further experimental validation.

Altogether, JCAD may represent a responsive element to mechanical stimuli and/or structural injuries to the cytoskeleton and cell-to-cell junctions. Although the biochemical function of JCAD is only partly understood, it likely regulates apoptosis, cell proliferation, differentiation, and response to hypoxia by interacting with multiple intracellular pathways. The biological consequence of JCAD activation appears context-dependent, varying with the upstream stimulus and the endothelial microenvironment; how each pathway is differentially engaged across distinct disease settings is discussed, based on the available evidence, in the sections that follow. Lastly, in the absence of structural data, the molecular interactions described should be regarded as functional associations that may involve adaptor-mediated or indirect mechanisms, formal structural characterization is required.

## 3. JCAD and cardiovascular diseases

Accumulating evidence implicates JCAD in cardiovascular disease through convergent effects on endothelial function, inflammation, and mechanotransduction.

Genetic evidence further supports JCAD’s involvement in cardiovascular pathology. Several genome-wide association studies have identified genetic variations within the JCAD locus, such as rs3739998, rs2505083, rs2487928, and rs9337951 (the latter identified as the lead SNP in the CARDIoGRAMplusC4D and UK Biobank meta-analysis, mapping to increased JCAD expression in aortic tissue by eQTL analysis), that are associated with an increased risk of coronary artery disease and acute coronary syndromes (ACS).^3–6^ Yet, the functional effect of these polymorphisms remains to be determined.

Mechanistic studies provide mechanistic support for these associations; *Jcad−/− ApoE−/−* mice exhibit reduced atherosclerosis and improved endothelial function. Specifically, the absence of *Jcad* in these mice led to a reduction in high-fat-diet-induced atherosclerosis and improved endothelium-dependent relaxation mediated by acetylcholine.^28,29^ These protective effects are largely attributed to a reduced inflammatory response observed in both animal models and human ECs lacking *JCAD*. Mechanistically, JCAD has been shown to stimulate the inflammatory cascade in ECs, facilitating monocyte recruitment and atherosclerotic plaque formation. Conversely, decreased JCAD expression has been linked to the downregulation of pro-atherogenic genes, such as vascular cell adhesion molecule 1 (VCAM-1), while upregulating genes with protective effects against atherosclerosis.^28^ Additionally, the inflammatory effects of *JCAD* are mediated through the Hippo pathway in a LATS2-dependent manner, as evidenced by the upregulation of endothelial JCAD expression in human atherosclerotic plaques.^28^ Furthermore, the spatial distribution of atherosclerotic lesions in *Jcad*−*/*− animals—significantly reduced in the aortic root and the inner curvature of the aortic arch—suggests a role for JCAD in mechano-transduction pathways. This is corroborated by *in vitro* studies in human ECs subjected to acute flow changes and *in vivo* experiments involving hind limb ischaemia, a model of altered blood flow dynamics.^28,29^ Whether JCAD-mediated PI3K/Akt inhibition extends to impaired eNOS activation remains to be established. Akt phosphorylates eNOS, a modification required for full enzymatic activation and nitric oxide production.^46^ Reduced Akt phosphorylation in response to acute shear stress has been reported in JCAD-silenced endothelial cells,^29^ raising the possibility that JCAD suppression of Akt could indirectly reduce eNOS-dependent NO bioavailability in the atherogenic endothelial microenvironment, contributing to impaired vasorelaxation beyond the inflammatory effects already described. In this context, integrin-linked kinase (ILK) represents a potential point of convergence: ILK preserves eNOS coupling and NO production in the vascular endothelium^47,48^ and shares functional overlap with JCAD in endothelial mechanosensing.^49^ Whether JCAD and ILK interact or regulate eNOS activity through convergent mechanisms has not been investigated. Direct experimental testing of this hypothesis is needed before any mechanistic conclusions can be drawn.

Beyond its role in atherosclerosis, JCAD contributes to arterial thrombosis, a key driver of myocardial infarction and ischaemic stroke. In experimental models, *Jcad−/−* mice displayed reduced thrombus formation following photochemically induced endothelial injury, suggesting a pro-thrombotic function for JCAD. At the mechanistic level, JCAD influences coagulation and fibrinolysis. The absence of JCAD led to decreased tissue factor (TF) expression and activity, resulting in reduced coagulation activation. Additionally, *Jcad−/−* mice exhibited increased fibrinolysis due to lower plasminogen activator inhibitor-1 (PAI-1) expression. These effects were further validated in human aortic ECs, where JCAD knockdown via small interfering (si)RNA suppressed TF and PAI-1 expression. Unlike its role in inflammation, this pro-thrombotic effect was not mediated by the Hippo pathway but rather through the PI3 K/Akt signalling cascade.^34^ Notably, pharmacological inhibition of PI3K with wortmannin restored the pro-thrombotic phenotype both in vivo and in vitro.^34^ As a preliminary clinical observation, plasma levels of JCAD were higher in patients with ST-elevation myocardial infarction as compared to those with chronic coronary syndrome and positively correlated with circulating TF and PAI-1.^34^ Of note, the cellular origin and precise biological significance of circulating JCAD remain to be fully characterized; mechanistic inferences from plasma levels to endothelial biology should therefore be interpreted with appropriate caution.

Consistent with these observations, we recently demonstrated in two large prospective ACS cohorts that circulating JCAD is not only mechanistically involved in atherothrombosis but also serves as an independent predictor of cardiovascular events in patients at high risk.^50^ In the SPUM-ACS cohort (*n* = 4787), JCAD plasma levels were strongly and independently associated with 1-year major adverse cardiovascular events (MACE) across patients with residual lipid risk, residual inflammatory risk, or both. In these groups, each doubling of JCAD plasma concentrations translated into a 27–47% increase in MACE risk, independent of low-density lipoprotein cholesterol (LDL-c), high-sensitivity C reactive protein (hs-CRP), age, sex, and other risk factors.^50^ These associations were consistent across all multivariable-adjusted models tested and exceeded the predictive contributions of LDL-c and hs-CRP within the same risk strata. In the external validation cohort (RISK-PPCI study; *n* = 496), plasma JCAD correlated well with indices of impaired endogenous fibrinolysis—including TF, thrombin activatable fibrinolysis inhibitor, PAI-1, and lysis time, implying a direct link between JCAD and prothrombotic pathways in patients with a recent ACS. Importantly, aligning with outcome data in SPUM-ACS, elevations in plasma JCAD levels predicted 1-year MACE, validating its prognostic value in an independent ACS population.^50^

In conclusion, *JCAD* plays a multifaceted role in regulating critical biological processes in ECs within the cardiovascular system, influencing cell behaviour, angiogenesis, inflammation, atherosclerosis, and thrombosis (*Figure 2*). Its dysregulation can have significant implications for various diseases related to endothelial dysfunction and vascular disorders.^34,50–52^ These findings suggest that *JCAD* represents a promising biomarker candidate and potential therapeutic target for cardiovascular disease, pending further clinical validation.^53^

**Figure 2:**
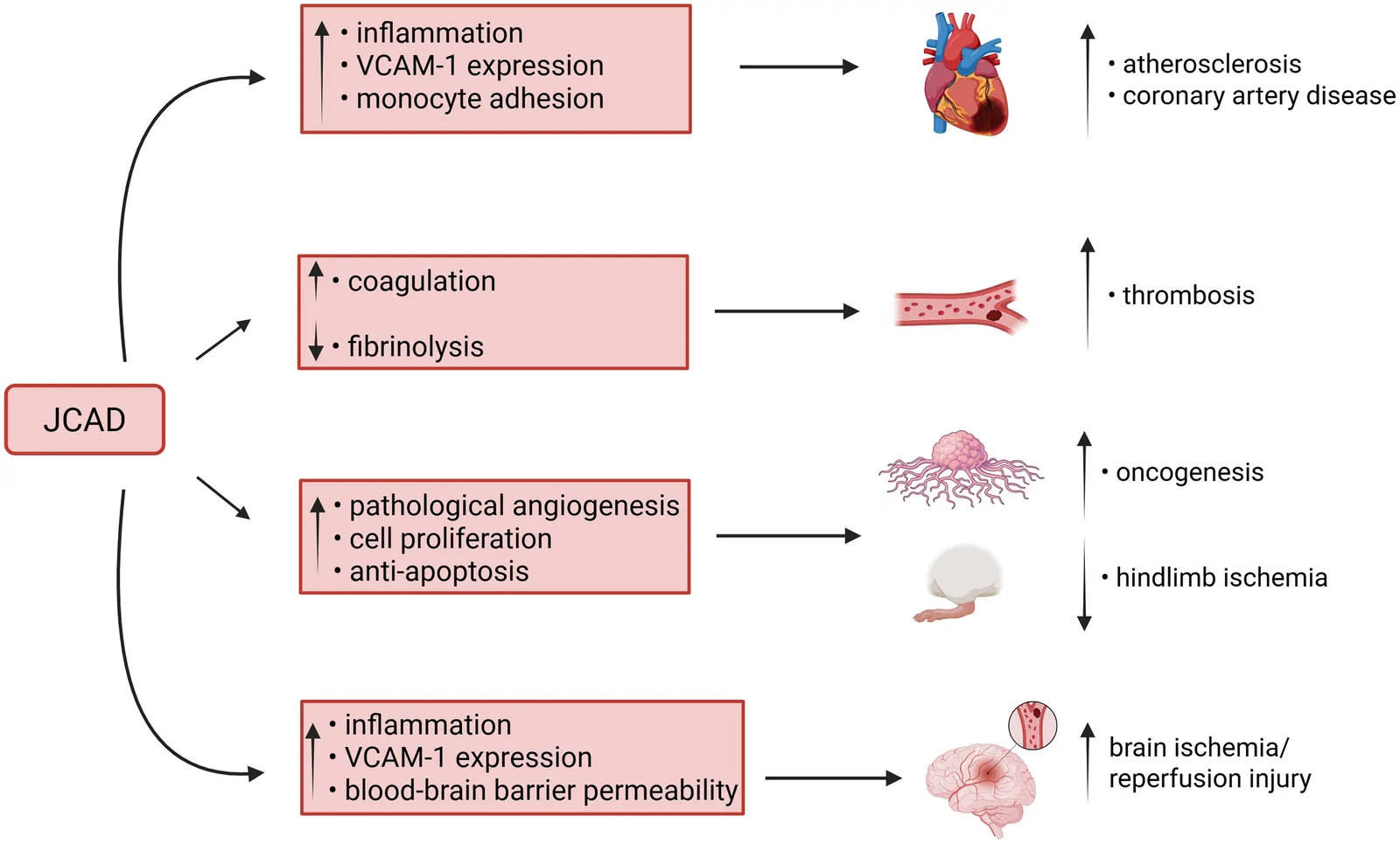
The role of JCAD in cardio- and cerebrovascular conditions. Recent evidence links JCAD expression to various cardiovascular and cerebrovascular conditions. By modulating inflammatory mediators, enhancing VCAM-1 expression, and promoting monocyte adhesion, JCAD contributes to the development of atherosclerosis and coronary artery disease. In the context of major cardiovascular events, JCAD plays a role in arterial thrombosis by increasing coagulation factors while reducing fibrinolysis, thereby disrupting the balance between clot formation and dissolution. Additionally, JCAD influences angiogenesis under pathological conditions, facilitating revascularization after hind-limb ischaemia but also promoting tumour growth. Finally, JCAD appears to contribute to cerebral ischaemia/reperfusion injury by exacerbating inflammation and blood–brain barrier damage, further highlighting its impact on vascular pathophysiology. JCAD, junctional cadherin 5 associated; VCAM-1, vascular cell adhesion molecule-1. Created in BioRender. Montecucco, F. (2026) https://BioRender.com/cdn6r1q.

## 4. JCAD and ischaemic stroke

Interest in a potential role of JCAD in cerebral vascular biology predates direct experimental investigation: the KIAA1462 locus was identified as a candidate locus for late-onset Alzheimer's disease in APOE4 carriers,^11^ and JCAD has been identified as a marker of human brain capillary endothelial cells in a large-scale single-nucleus transcriptomic atlas of the human brain vasculature,^54^ providing biological plausibility for the experimental studies described below. We recently investigated the role of JCAD in acute ischaemic stroke and found that its endothelial expression negatively impacts stroke outcomes by increasing blood–brain barrier (BBB) disruption and promoting vascular inflammation, both of which are critical in the progression of ischaemic brain damage (*Figure 2*).

In murine models of ischaemic stroke, the selective deletion of *Jcad* in endothelial cells led to a reduction in ischaemic lesion size and an improvement in neurological function, suggesting that JCAD aggravates stroke severity. This was confirmed in experiments where *Jcad−/−* mice, as well as endothelial-specific JCAD knockout (*eJcad*−/−) mice, exhibited smaller ischaemic volumes compared to control groups. Post-ischaemic silencing of JCAD using siRNA—mimicking the situation of patients with ischaemic stroke and being treated with thrombolytic and anti-JCAD drugs—similarly improved outcomes, reducing both ischaemic damage and BBB permeability. These findings suggest that JCAD plays a detrimental role during the reperfusion phase following stroke, making it a potential target for therapeutic intervention.^30^

Mechanistically, JCAD appears to mediate its effects in ischaemic stroke through the PI3 K/Akt signalling pathway. After ischaemia-reperfusion injury, JCAD prevents the activation of Akt, a protein kinase that promotes cellular survival and reduces inflammation. By inhibiting Akt phosphorylation, JCAD leads to increased cellular death and vascular inflammation, as evidenced by the upregulation of VCAM-1 in ECs following hypoxia/reoxygenation injury with detrimental effects on BBB preservation.^30^

Such experimental observations were reproduced in vitro in human brain microvascular ECs exposed to hypoxia/reoxygenation, where JCAD silencing preserved endothelial barrier integrity and reduced cell death and VCAM-1 expression, indicating that JCAD contributes to endothelial dysfunction during ischaemic events.^30^

Beyond animal models, JCAD also holds potential as a biomarker in human patients with ischaemic stroke. Indeed, elevated levels of circulating JCAD were observed in stroke patients and were associated with worse clinical outcomes, including higher 90-day mortality rates independently of established risk factors.^30^ Also, we reported the association between circulating JCAD and assessed by C-reactive protein levels, with mediation analysis suggesting systemic inflammation as the mediator of the association between JCAD and mortality. These findings corroborate our experimental findings and underscore the potential translational relevance of JCAD as both a prognostic biomarker and a therapeutic target.^30^

## 5. Outlook

It is important to note that the following paragraphs present hypothesis-generating extrapolations based on JCAD’s known biology, pending direct experimental evidence. Given its important role in the regulation of endothelial junctions, vascular integrity and inflammation, JCAD emerged as an important determinant of endothelial dysfunction. Besides atherosclerosis, and its sequelae such as ACS and ischaemic stroke, endothelial dysfunction is a well-established precursor to many CV conditions in which JCAD may play a role.

Genetic studies have reported a significant relationship between JCAD polymorphisms and blood pressure regulation,^55^ providing a biological rationale for exploring a potential role of JCAD in hypertension. Given its established involvement in endothelial mechanotransduction and vascular inflammation, it is conceivable that JCAD dysregulation could contribute to altered vascular tone, though direct experimental evidence supporting this hypothesis is currently lacking. Of note, loss of *Jcad* does not affect blood pressure or heart rate in knockout mice under standard conditions,^29^ indicating that any putative contribution to hypertension is likely context-dependent and may require additional metabolic or haemodynamic stressors to become apparent. The Hippo pathway, one of the established downstream targets of JCAD, has independently been implicated in hypertension,^56^ providing further indirect biological plausibility. Further studies are needed to elucidate whether and through which mechanisms JCAD contributes to blood pressure regulation.

Similarly, the Hippo pathway is an important modulator of cardiac remodelling with proven roles in heart failure.^56^ Again, endothelial dysfunction is centre stage here, especially in heart failure with preserved ejection fraction, in which microvascular dysfunction has been identified as a defining characteristic.^50,57^ JCAD may once again represent a therapeutic target here through its role in increased oxidative stress, and inflammation, all contributors to myocardial stiffness and diastolic dysfunction.^58^ Beyond classical vascular disease, JCAD may also intersect with the recently defined cardiovascular–kidney–metabolic syndrome (CKMS).^59^ Indeed, CKMS is a multifaceted condition integrating cardiovascular, renal, and metabolic dysfunctions, often driven by hypertension and heart failure. Endothelial dysfunction is a common thread linking the cardiovascular and renal systems in CKMS.^60^ In addition to the vascular effects, JCAD may influence the progression of this syndrome by impairing glomerular filtration and facilitating albuminuria, which are early markers of renal involvement in CKMS. Beyond the cardiovascular system, recent evidence further extends JCAD’s biological relevance: JCAD loss has been shown to attenuate acute lung inflammation and emphysema in murine COPD models,^61^ and to reduce hepatic stellate cell activation and cholestatic fibrosis via the Hippo-YAP axis.^62^ These findings suggest that JCAD may function as a broader regulator of inflammation across multiple organ systems, an observation with potential relevance in the context of CKMS.

Lastly, a speculative but biologically plausible role for JCAD in calcific aortic valve disease (CAVD) may also be considered. CAVD initiates at the endothelial surface of the aortic valve leaflets, where disturbed flow promotes endothelial activation and, through endothelial-to-mesenchymal transition (EndoMT), the acquisition of an osteogenic phenotype by valvular endothelial cells.^63^ YAP/TAZ signalling has been implicated in mechanosensing and valvular mineralization in this context,^63,64^ and given JCAD's established role in promoting YAP nuclear translocation in response to disturbed flow, it is conceivable that JCAD could act as an upstream facilitator of these processes. This hypothesis remains entirely speculative in the absence of direct experimental evidence, but warrants investigation given the unmet therapeutic need in CAVD.

## 6. Knowledge gaps and therapeutic challenges

Several limitations should be acknowledged in the context of current knowledge. The structural and biochemical characterization of JCAD remains incomplete: no functional domains have been resolved, and the molecular basis of its interactions with downstream signalling partners has not been formally established. The functional consequences of the CAD-associated genetic variants (rs9337951, rs2487928, and others) remain largely uncharacterized at the protein level, and the mechanism by which genetic risk translates into altered JCAD biology awaits clarification. The mechanistic evidence base derives predominantly from murine atherosclerosis models and cultured human endothelial cell lines. The circulating biomarker evidence, while promising, would benefit from independent replication in diverse populations, assay standardization, and head-to-head comparison with established risk markers. Finally, JCAD may exert context-dependent effects across different vascular beds, disease stages, and cell types (including non-endothelial cells where its expression has been documented) which may influence the generalizability of current findings.

These biological uncertainties translate directly into substantial challenges for therapeutic translation. No specific agonists or antagonists targeting JCAD are currently available. JCAD is a scaffolding protein with no canonical enzymatic domain, which makes direct pharmacological inhibition with conventional small molecules considerably more difficult. The indirect approaches identified to date carry significant limitations: (i) the broad-spectrum BET bromodomain inhibitor JQ1 reduces JCAD expression through compound screening,^28^ but its effects are pleiotropic and cannot be attributed specifically to JCAD modulation; (ii) miR-19a-3p downregulates JCAD expression in human endothelial cells by targeting its 3'-UTR, reducing YAP/TAZ nuclear localization and monocyte adhesion,^65^ but miRNA therapeutics face well-recognized delivery and off-target challenges in the vascular context. A further concern is the oncogenic safety liability of systemic JCAD inhibition: JCAD promotes pathological angiogenesis^66^ and tumour progression in hepatocellular carcinoma,^67^ and sustained suppression could theoretically impair tissue repair and interfere with tumour surveillance. Finally, a clear conceptual distinction must be maintained between JCAD as a biomarker candidate, a role supported by current literature, and as a validated therapeutic target, which would require formal demonstration that JCAD-specific intervention modifies CV outcomes in humans.

## 7. Conclusion

JCAD/KIAA1462 has emerged as a biologically plausible endothelial mediator across a spectrum of cardiovascular and ischaemic cerebrovascular conditions. Converging genetic, experimental, and clinical evidence supports its involvement in endothelial dysfunction, atherosclerosis, arterial thrombosis, and acute ischaemic stroke, with circulating JCAD showing early promise as a biomarker of residual thrombotic risk. The experimental evidence is robust and early clinical data are promising, whether these findings will ultimately inform therapeutic strategies remains an open question worthy of investigation.

## Data Availability

No new data is presented in this review article.
